# Supplementary material for: Terahertz Magnon-Polariton Control Using a Tunable Liquid Crystal Cavity
Source: ACS Photonics. 2025 Dec 5;12(12):6762–9. doi: 10.1021/acsphotonics.5c01879 (PMC12715831; doi:10.1021/acsphotonics.5c01879)
Supplement: Supplementary file 1 [file ph5c01879_si_001.pdf]

Supplementary Information: Terahertz magnon-polaritons control using a tunable liquid crystal cavity

Dmitriy Yavorskiy,[1,2,3] Jan Suffczyński,[4] Rafał Kowerdziej,[5]
Olga Strzeżysz,[6] Jerzy Wróbel,[2,5] Wojciech Knap,[1,3] and Marcin Białek[1,*]

[1] *Institute of High Pressure Physics, Polish Academy of Sciences, Sokołowska 29/37, 01-142 Warsaw, Poland*
[2] *Institute of Physics, Polish Academy of Sciences, Aleja Lotników 32/46, 02-668 Warszawa, Poland*
[3] *CENTERA, CEZAMAT, Warsaw University of Technology, Poleczki 19, 02-822 Warsaw, Poland*
[4] *Institute of Applied Physics, Military University of Technology, Kaliskiego 2, 00-908 Warsaw, Poland*
[5] *Institute of Experimental Physics, Faculty of Physics, University of Warsaw, Pasteura 5, 02-093 Warsaw, Poland*
[6] *Institute of Chemistry, Military University of Technology, Kaliskiego 2, 00-908 Warsaw, Poland*

(Dated: October 14, 2025)

* marcin.bialek@unipress.waw.pl

S1. TIME-DOMAIN REFLECTION SPECTRA

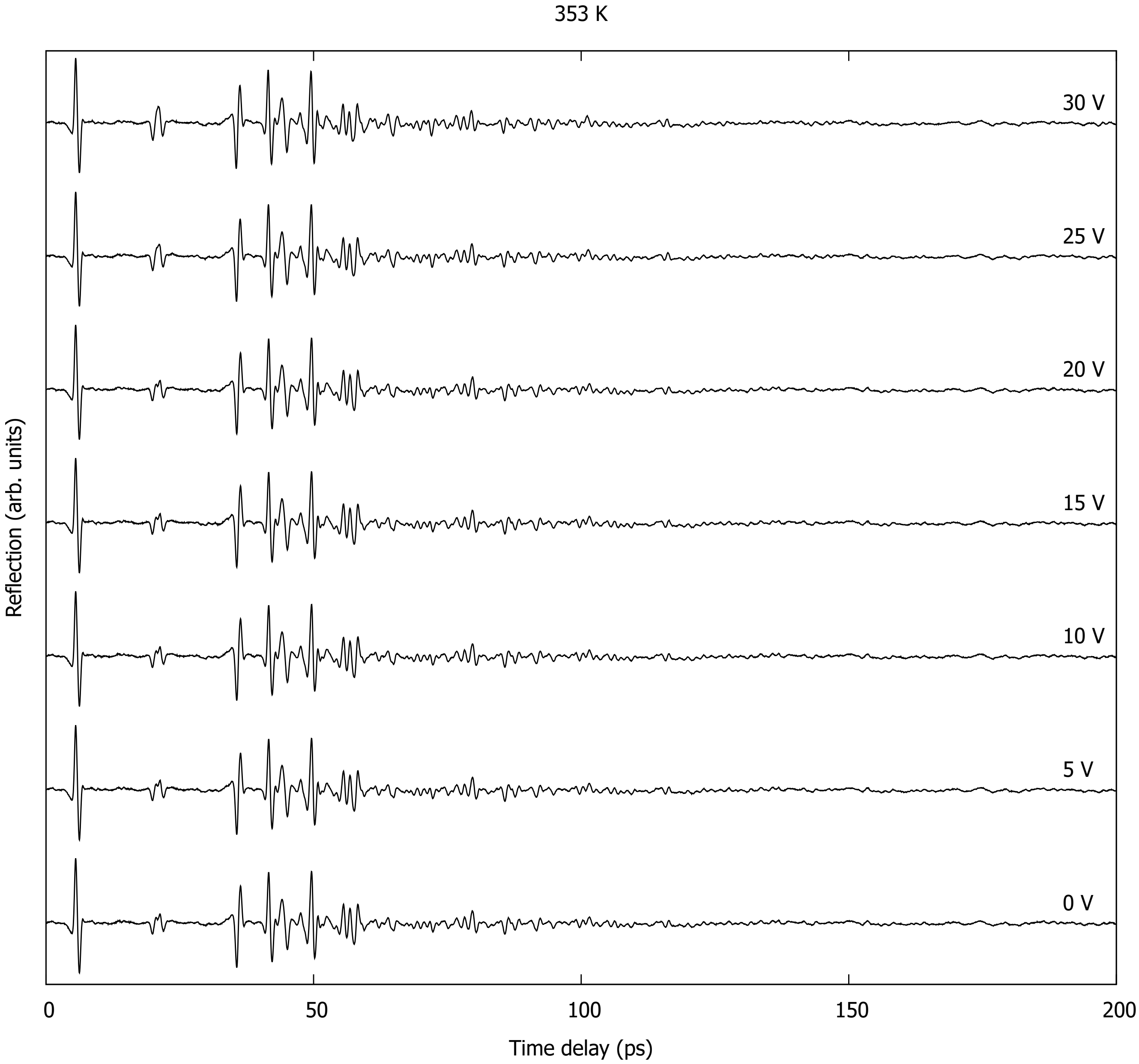

Figure S1. Raw time-domain reflection traces measured at different voltage biases. From left to right, the reflections correspond to consecutive interfaces: air–silica, silica–liquid crystal–silica, silica–air, air–NiO, and NiO–air, followed by the second-order internal reflection.

S2. NIO MAGNON RESONANCE.

Coupling with Fabry–Perot cavity modes enhances the amplitude of the AFM magnon resonance through the formation of magnon–polariton modes. In a parallel-plane slab, the Fabry-Perot modes are formed naturally; therefore, with our samples, it's difficult to observe a magnon not interacting with cavity modes. The closest approximation of a pure magnon is when the magnon has a frequency strongly detuned from cavity modes. We present below two reflection spectra collected from the same sample as used in the experiment described in the manuscript. These are for two different temperatures, at 342 K magnon is tuned with a Fabry-Perot mode at about 0.95 THz, and the amplitude of magnon-polaritons reaches about 80%. In contrast, at 392 K, the magnon at about 0.89 THz is detuned from cavity modes; the peak amplitude reached about 5%. Thus, we estimate the amplitude enhancement to about 16.

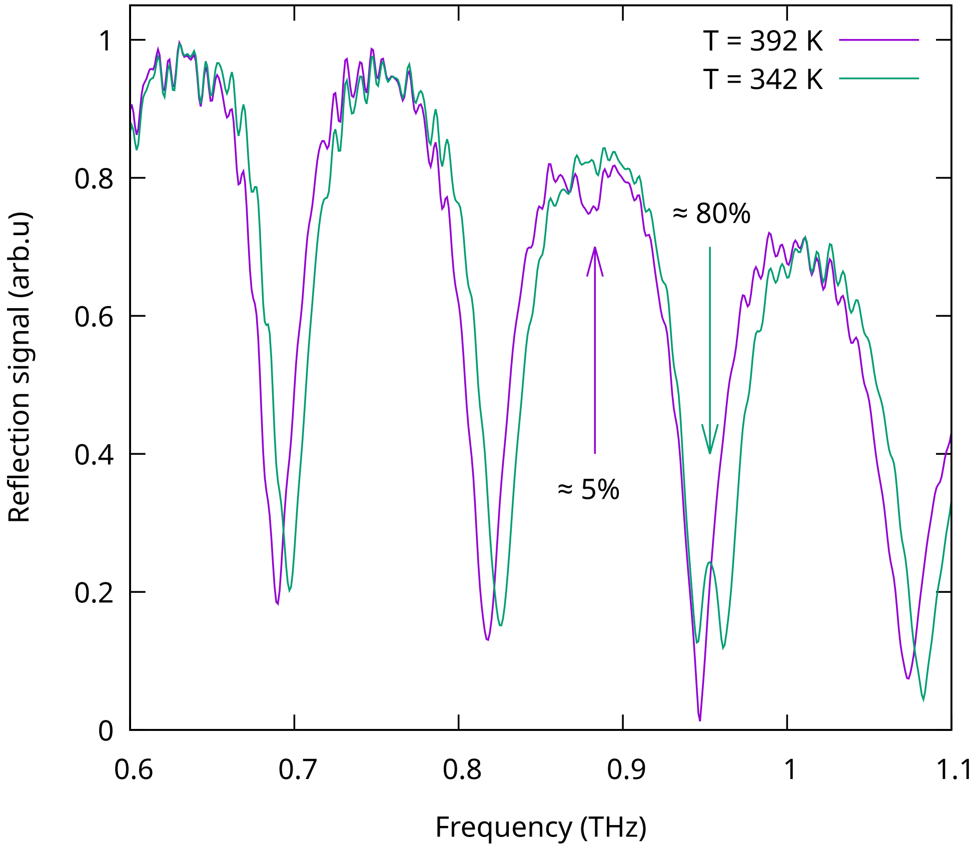

Figure S2. Reflection spectra vs frequency at two different temperatures. The Fabry–Perot cavity mode coupled to the magnon and the uncoupled magnon resonance are indicated with purple and green arrows, respectively.

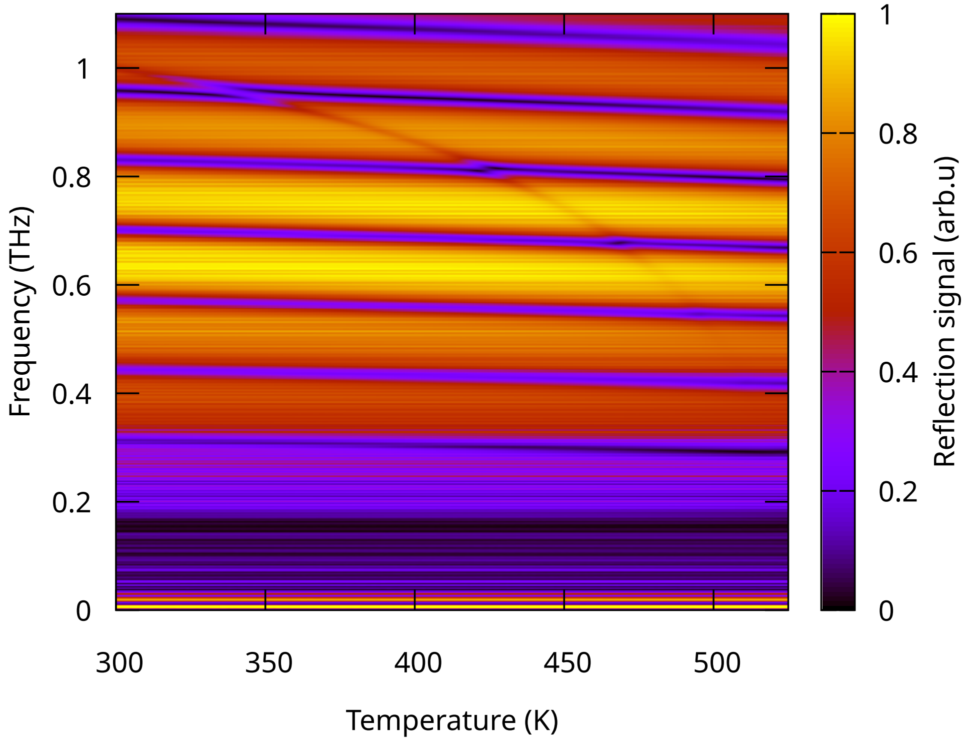

Figure S3. Temperature dependence of reflection spectra measured from the NiO 330-μm-thick samples alone. Measurements were performed at an 8-degree incidence angle, in contrast to 0 degrees in the manuscript.

S3. SPECTRA DE-CONVOLUTION.

We have fitted Lorentzian curves to both the uncoupled cavity mode number 3 and the uncoupled magnon. To extract the linewidth of the uncoupled cavity mode, we performed the fitting at T = 295 K, where the magnon resonance is shifted above 1 THz and therefore does not interact with the considered cavity modes. Under these conditions, we obtained full widths at half maximum (FWHMs) of the P_1, P_2, and P_3 modes, respectively, of 6.1 GHz, 7.4 GHz, and 6.6 GHz.

For the uncoupled magnon, we use the FWHM obtained from measurements on a bare NiO sample, as reported in Ref. 27 [M. Białek et al., Adv. Funct. Mater. 35, 2416037 (2025)], γ_M = 8.0 GHz ≈ parameter of the Lorentzian function describing antiferromagnetic resonance in magnetic susceptibility.

Taking the linewidths of the uncoupled magnon and the P_2 cavity mode as defined by their FWHM values, we compare their mean value, $(\gamma_{P2} + \gamma_M)/2 = 7.7$ GHz, with the interaction strength $\Omega_2 = 11.2$ GHz at 40 V (see Fig. 5(d) in the manuscript). Since Ω_2 $(\gamma_{P2} + \gamma_M)/2$, these calculations quantitatively confirm that the system remains in the strong coupling regime.

Based on the linewidth and spectral position of a given resonance, we determined the Q-factor for each cavity mode that is not interacting with the magnon. For all considered cavity modes, the Q-factors are in the range 130-160.

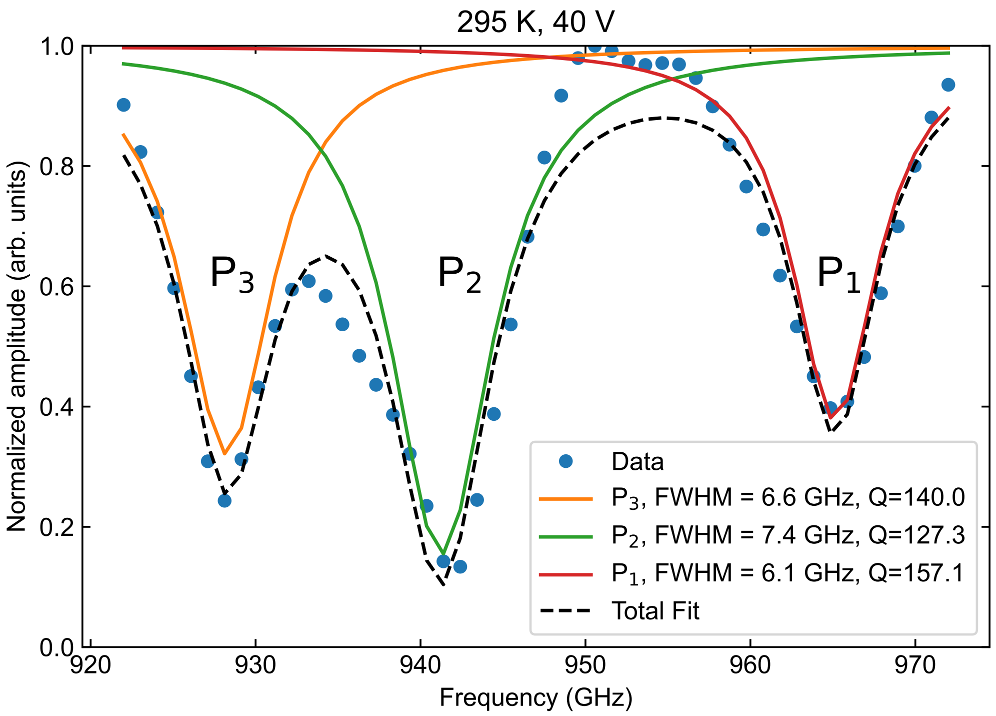

Figure S4. Reflection spectrum (blue dots) measured at 295 K and the voltage bias of 40 V in the spectral range of the P1, P2, and P3 modes, together with a fit (black dashed line) using a sum of three Lorentzian curves (colored solid lines). The label indicates the linewidths and Q-factor values determined from the fit.

S4. VOLTAGE BIAS EFFECT ON CAVITY MODES

We have extracted the reflection spectra of the LC cell without the NiO layer by applying an FFT window up to 40 ps (i.e., before the pulse reaches the NiO). This eliminates the NiO-related features from the Fabry–Perot cavity mode. As shown below in Figure S5, reflection of the LC cell results in a different set of cavity modes, which are temperature-independent, proving that in our system, the temperature of the LC cell did not increase substantially when NiO was hot.

We have also excluded the magnon contribution from the reflection spectra by applying a window function to the FFT calculation, so that only the refraction from the liquid crystal cell and the NiO layers are taken into account, while the magnon excitation is suppressed. The results of such a calculation are shown in the figure below. It is interesting to compare this result with the FFT of LCC only (Fig. S5), which does not exhibit any temperature dependence.

Below, we have presented figures showing reflection registered at 300 K versus applied voltage for consecutive frequency ranges as color maps. The liquid crystal enables tuning of the cavity modes from very low frequencies up to 1.8 THz.

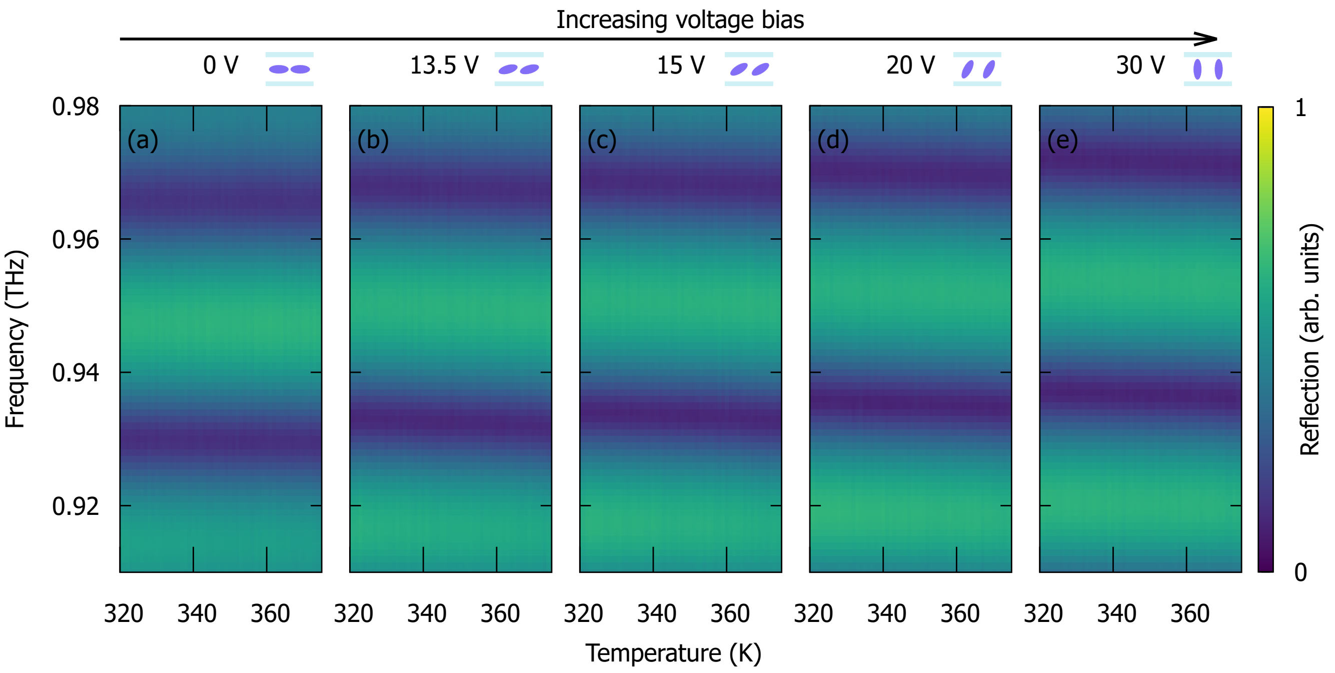

Figure S5. Reflection spectra as a function of temperature for consecutive voltage biases in the range from 0 V to 30 V. The FFT is windowed in the temporal domain to the region corresponding solely to the reflection from the liquid-crystal cell without reflections from the NiO crystal.

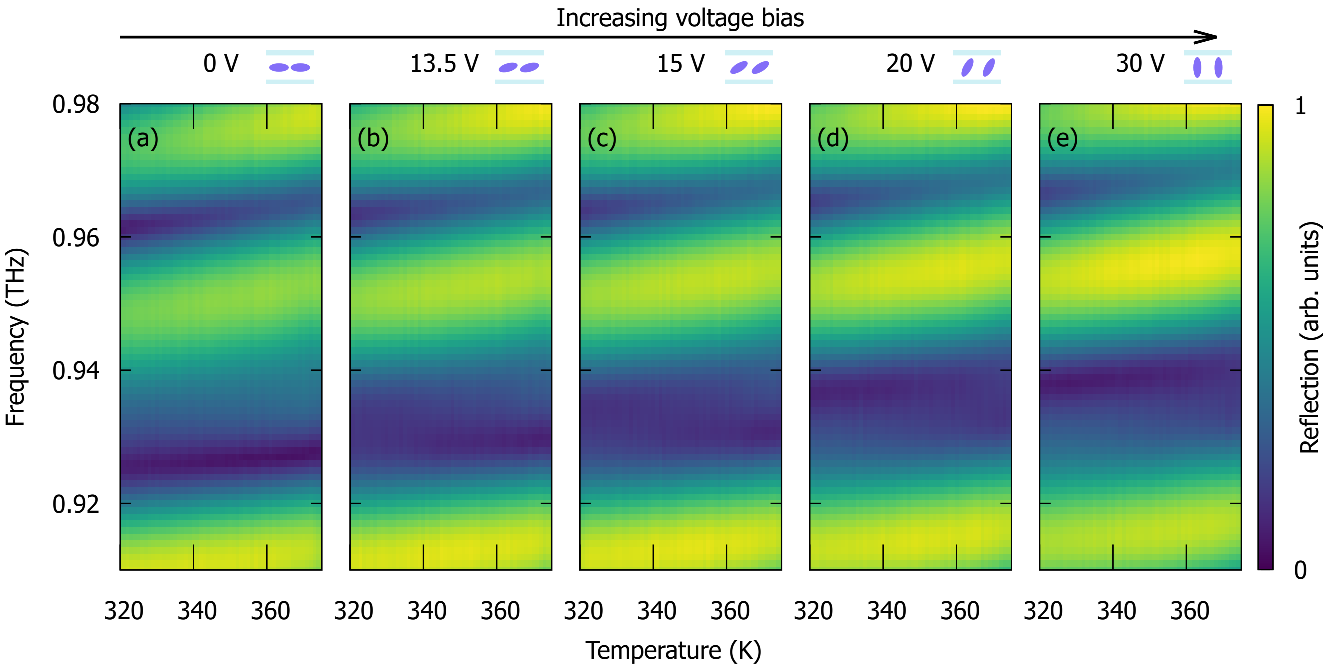

Figure S6. Reflectivity spectra as a function of temperature for consecutive voltage biases in the range from 0 V to 30 V. The FFT is windowed in the temporal domain range to peaks related to reflections from the interfaces of the liquid-crystal cell and NiO, without subsequent echoes and oscillations related to excitations of the magnon.

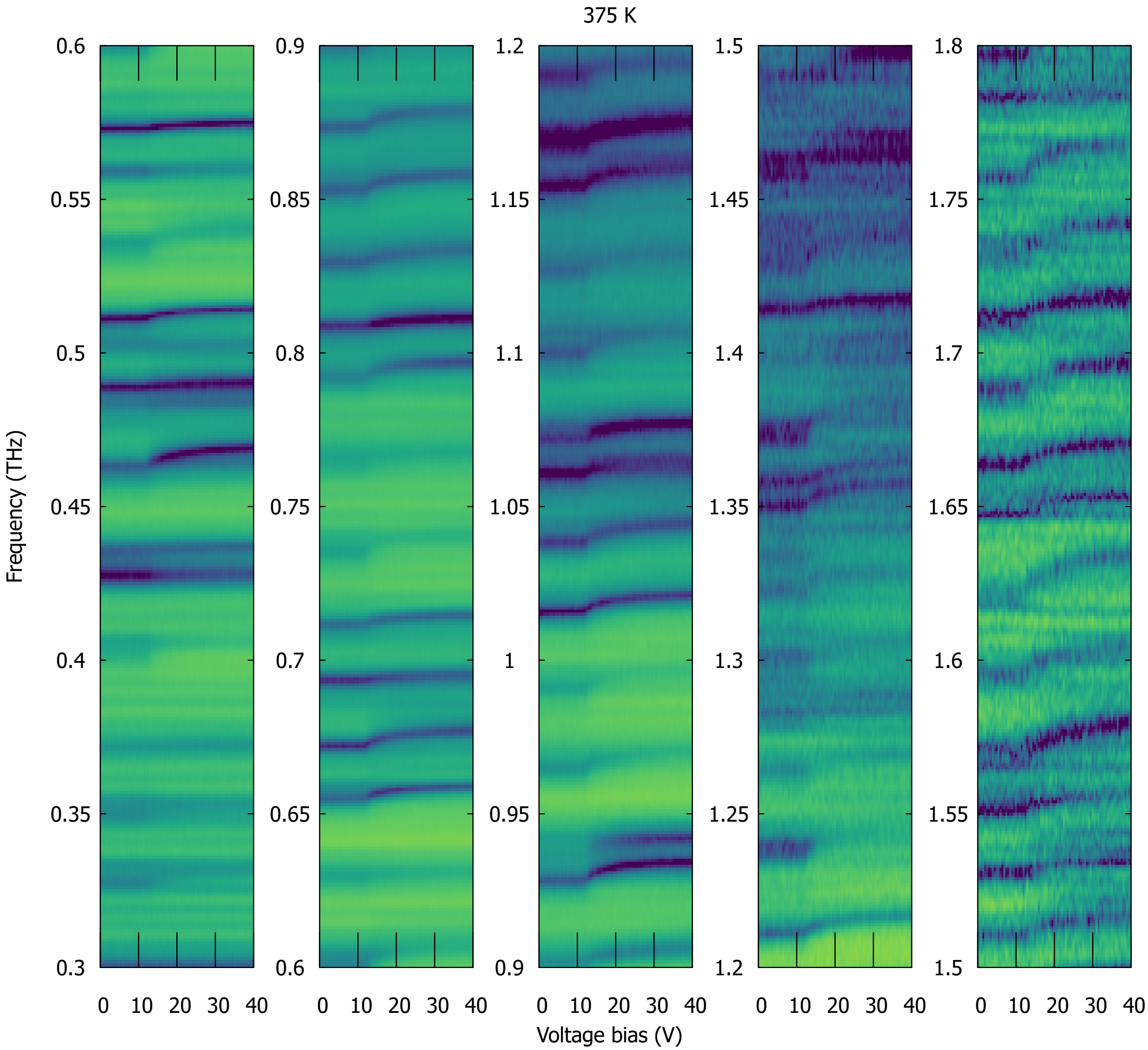

Figure S7. Reflection in consecutive frequency ranges of the structure comprising NiO layer and a liquid crystal cell as a function of voltage bias applied to the liquid crystal cell at 353 K.
